# Validation of PurpleAir PM_2.5_ Sensors in Temperate Urban Environments

**DOI:** 10.1021/acsestair.5c00443

**Published:** 2026-09-18

**Authors:** Charles C. Sylvester, Nathaniel D. Hauenstein, Kabindra M. Shakya

**Affiliations:** Department of Geography and the Environment, 8210 Villanova University, 800 Lancaster Avenue, Villanova, Pennsylvania 19085, United States

**Keywords:** air pollution, low-cost sensor, accuracy, precision, community monitoring, Philadelphia

## Abstract

Low-cost sensors (LCS), such as PurpleAir (PA), are widely used for measuring fine particulate matter (PM_2.5_) because they are inexpensive, easy to deploy, and provide publicly accessible data. However, PA sensors are known to overestimate PM_2.5_, and thus, several correction methods have been developed to improve their accuracy. In this study, we evaluated accuracy by comparing nationwide and locally derived correction methods during summer and spring seasons, examined PA output data types, assessed the influence of relative humidity using both PA and weather station data, and analyzed the performance after one year of field deployment. In the moderate climate of Philadelphia, the nationwide correction of Barkjohn et al. (2021) combined with using the mean value of two PA_atm_ data outputs (RMSE = 1.640 μg/m^3^, MBE = 0.103 μg/m^3^, *R* ^2^ = 0.683) for both 1 h and 24 h measurements provided the highest accuracy. Relative humidity measurements, whether obtained from a weather station or the PA sensor, had minimal effect on PM_2.5_ accuracy (difference in *R* ^2^ = 0.04). PA sensor accuracy declined after one year of use (*R* ^2^ = 0.567), showing requiring extra sensor calibration in long-term deployment. This methodology enhances the performance of PA sensors and establishes greater trust in the LCS.

## Graphical abstract

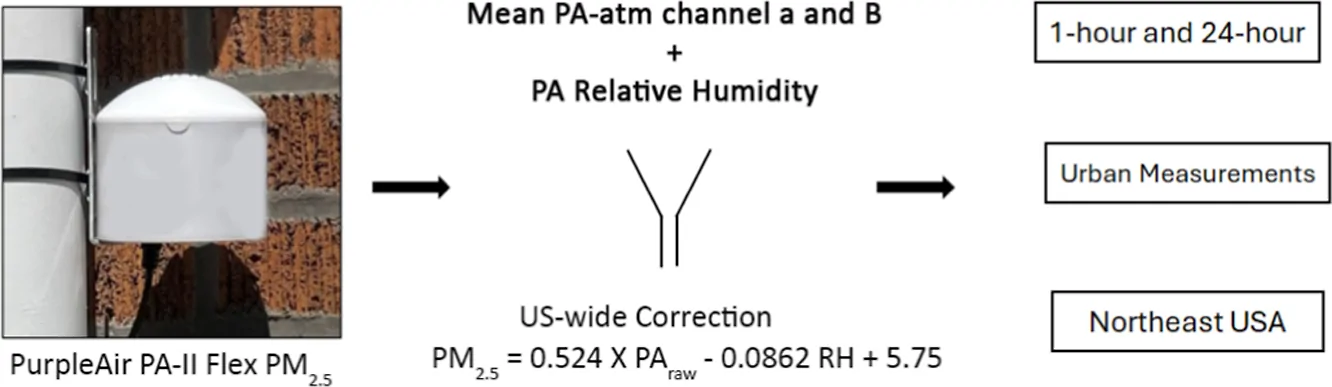

## 1 Introduction

The recent development of low-cost sensors (LCS) to monitor fine particle air pollution (PM_2.5_: particles smaller than 2.5 micrometers in diameter) has increased the ability to monitor PM_2.5_ and increased data accessibility of air quality.^1–3^ In contrast to the limited number of central monitoring stations sparsely scattered across the United States (US), LCS have enabled community-scale air quality monitoring with fine spatial and temporal resolution. By definition, LCS are inexpensive, easy to operate, require minimal maintenance, and some, such as PurpleAir (PA), make data freely available to the public. They have significantly enhanced public awareness of local air quality and empowered individuals to participate in citizen science by installing their own LCS. Further development of LCS is imperative so that disproportionately burdened communities can have accurate air quality information that will inform future land development and policy decisions.^4^

PA sensors are a small, common, optical-based LCS for measuring PM_2.5_ with a network of over 35,000 monitors worldwide at a relatively low cost (<US$300).^5^ They require a power connection, and when connected to Wi-Fi, PA sensors report data to *map.purpleair.com* for public data dissemination. PA provides PM_2.5_ readings every 2 min in three output types: atm, cf_1, and alt, which are generated from different algorithms that the optical particle counter uses to convert the light scattering into particle count and size.^6^ PA recommends using the output type of atm for ambient air monitoring; however, there is not a clear consensus in the scientific community due to deviations, which creates uncertainty that must be clarified.^7–10^

LCS must be validated to ensure scientific reliability and trustworthiness. PA tends to overestimate PM_2.5_ measurements.^11,12^ Many approaches have addressed the overestimation by PA, but further improvement and validation with the newest models of PA sensors, such as the PA-II Flex model, and evaluation of performance after its field deployment are needed. Several relative humidity (RH)-based data corrections have been proposed to improve PA PM_2.5_.^13–16^ These corrections address the errors caused by hygroscopic growth of particles in the optical particle counters. The drawback of nationwide data correction methods is that RH is extremely variable across climatic regions, and the use of nationwide corrections must be validated in different geographic areas as results have varied in their effectiveness.^17–19^ This has led to lower confidence in previous correction methods. Additionally, uncertainty remains regarding the accuracy associated with the source of RH used in the correction equation. It would be ideal to use RH measurements from the PA sensor itself; however, concerns remain regarding the accuracy of PA RH, especially for RH greater than 40%.^20,21^ Therefore, a valid alternative to account for the PA PM_2.5_ overestimation is to develop a local linear PM_2.5_ correction either by collocating with a reference monitor or comparing to the nearest reference monitor (typically a government monitoring station).^7,11^ This technique generates a local PM_2.5_ correction, but that correction should only be used for a specific study. Nationwide and local corrections must be regionally validated for accuracy to make future research more effective. Finally, there is concern regarding the accuracy of PA PM_2.5_ measurements after sensor deployment over long periods of time, with PA advertising that sensors have a 2 year lifespan.^20^ Accuracy is shown to greatly degrade after years of use with older models.^22^ As new generations of PA are released with updated optical particle counters, accuracy from previous corrections may change along with their longevity. In addition to accuracy concerns, as more researchers use PA to explore spatial and temporal variation, the accuracy of each model of PA must be assessed.

This study addresses a critical gap in PA PM_2.5_ measurement by using the latest model of PA-II Flex across 1 h and 24 h time scales in urban and suburban experimental setups. This study aims to address five key research questions. (1) What is the optimal PA output data type for use (atm or cf_1 and individual or mean of two channels)? (2) How does the use of uncorrected PA RH, corrected PA RH, and RH from a local weather station affect PM_2.5_ accuracy? (3) Is there a difference between a data correction developed for nationwide use versus a simplified local correction method? (4) What is the difference in PA accuracy at the 24 h and 1 h time scales? (5) Does PA sensor performance—specifically accuracy and precision—deteriorate after one year of field deployment? By addressing these questions, this study aims to define the optimal method to implement one of many PA data correction equations that account for key factors influencing PA sensor performance in air quality research. This will enhance the accuracy and scientific reliability of PM_2.5_ measurements using PA sensors, supporting more robust community-level air quality monitoring.

## 2 Method

### 2.1 Experimental Setup

This study deployed PA-II flex air quality sensors, which are equipped with dual laser particle counters (Plantower PMS-6003) and a pressure, temperature, humidity, and gas sensor (Bosch BME688). All data were collected via the micro-SD logger within each PA sensor. Five different outdoor collocation periods across three locations were conducted for this study by comparing PM_2.5_ measured from PA sensors to a higher-grade reference monitor.

Each of the five sampling periods (Table 1) was chosen to assess PA accuracy under various conditions. The first Villanova University (VU) collocation allowed for many sensors to be tested in a single area so that a plethora of data corrections could be compared before deploying the sensors in West Philadelphia.^23^ The second Villanova colocation tested the accuracy of a subset of sensors after one year of deployment. The two West Philadelphia colocations allowed for comparison of one PA sensor in an urban setting over two seasons—spring and summer. Finally, the Northeast Philadelphia collocation was chosen so that the 1 h time scale could be tested in an urban setting (Table 1).

**Table 1:** Summary Table of Five Collocation Periods

location	type	reference	number of PA	date	duration (days)	time scale tested (h)
Villanova University Rooftop (4-storey building)	suburban	gravimetric (Tisch environmental)	16 new	June 2024	5	24
			5 old, 5 new	June–July 2025	11	24
West Philadelphia Rooftop (1-storey building)	urban		1 new	July 2024	28	24
			1 old (after 6 months of deployment)	March–April 2025	20	24
Northeast Philadelphia Rooftop (a trailer)	urban	continuous (Teledyne T640)	1 new	April–July 2025	97	1

### 2.2 Sampling

Four collocations (both VU and both West Philadelphia) deployed the gravimetric method for 24 h measurements. For gravimetric method measurements, PM_2.5_ particles were collected on pre-weighed 47 mm PTFE membranes at a 16.67 L/min flow rate using a Tisch Environmental Wilbur Low Volume Air Sampler (TE, EPA Designated Reference and Equivalent Methods for PM analysis: RFPS-1014-219 PM_2.5_). TE was calibrated in the field following procedures in its manual mode. All filters were conditioned for at least 24 h in a chamber at ∼24 °C and 30–45% humidity before pre- and post-weighing. The change in weight of the membranes after ∼24 h of sampling was measured using a microbalance (Mettler Toledo, XP2U model) equipped with an antistatic kit. Weights were validated by taking replicate measurements until three measurements agreed within one microgram.

At VU, sampling was conducted on the rooftop of a four-story building for 5 days during June 2024 and for 11 days during June-July 2025. During June 2024, 16 new, previously unused PA sensors were collocated with a TE sampler. We deployed the PA sensors in Philadelphia for community monitoring, and five PA sensors were brought back from the field after about a year of use and were collocated with the TE sampler during June–July 2025. It is reasonable to conclude that the five sensors were exposed to similar weather and pollution conditions because all sensors were deployed in West Philadelphia. The mean PM_2.5_ ranged from 7.07 to 9.41 μg/m^3^ during deployment.^23^ A subset of sensors were selected randomly.

The two West Philadelphia collocations evaluated accuracy by collocating one PA sensor with TE in an urban environment in West Philadelphia. During July 2024 (number of days = 28) and March–April 2025 (number of days = 20), PM_2.5_ particles were collected on 47 mm PTFE filters for 24 h periods using a TE sampler. In addition to VU and West Philadelphia, another new PA sensor was collocated at the Northeast Waste monitoring site managed by Philadelphia Air Management Service for three months, from April 15 to July 21, 2025 (number of days = 97) for the fifth collocation. This site deploys a Teledyne T640 at 5.0 LPM to continuously monitor for PM_2.5,_ which was compared hourly to the collocated PA.^24^ These two long-term sites were chosen to reliably evaluate the best correction method determined by the VU collocation results in a more variable urban environment at both the 1 h and 24 h time scales. A total of five evaluation periods were conducted: two at the Villanova site (summers 2024 and 2025), two in West Philadelphia (summer 2024 and spring 2025), and one at the Northeast Philadelphia site (spring through summer 2025).

Throughout both VU collocations, the average daily temperature was 25.8 °C, the average RH was 67.7%, with precipitation on 43.4% of days. During the West Philadelphia collocation, the most moderate weather was found across all experimental setups in this study, with an average daily temperature of 20.0 °C, a daily RH of 65.4%, and precipitation on 42% of days. Weather at the Northeast monitoring site had an average daily temperature of 21.6 °C, an average RH of 67.9%, and precipitation on 40.2% of days. The National Oceanic and Atmospheric Administration classifies Pennsylvania within the Northeast US climatic region.^25^

### 2.3 PurpleAir Data Collection and Cleaning

Two minute raw data reported by each PA sensor (both PA_atm_ and PA_cf_1_) was downloaded from the microSD card. One of the data inclusion criteria was that channels a and b (for both PA_atm_ and PA_cf_1_) agree within 5 μg/m^3^ or 61% (two standard deviations) of each other. The 2 min PM_2.5_ data was averaged to the hourly level with the stipulation that the sensor must have been active for at least 40 of the 60 min. Then, the hourly data was used for analysis. This resulted in 99.23% data completeness for the Villanova collocations (45 hourly records removed), 100% data completeness for the West Philadelphia collocations, and 99.70% for the collocation at the Philadelphia Air Management site (7 hourly records removed).

### 2.4 PM_2.5_ Correction Methods, Output Types, and Relative Humidity Data Sources

Two local linear data corrections were developed to test the regionally specific data corrections. Local linear corrections followed the formula in eqs 1 and 2 when raw PA PM_2.5_ was compared against the reference monitor to generate a correction factor that can be applied to future measurements (Table 2). These local linear corrections do not account for other variables such as temperature or humidity. The correction factors were independently developed before application to the West Philadelphia collocations. This study generated the correction factors during the VU experiments from (1) the collocation with the gravimetric method using TE and (2) the corresponding daily data from the nearest government monitoring site. The nearest monitoring station to VU is the Norristown A420910013 site, managed by the Pennsylvania Department of Environmental Protection. The sampling site deploys a Teledyne T640 continuous monitor for PM_2.5_.

**Table 2:** Correction Methods Used throughout Villanova University Collocation

number	method	equation
eq 1	simple correction with TE	${PM}_{cf}=\frac{{PM}_{ref}}{{PM}_{raw}}$
eq 2	simple correction with nearest government monitoring station	${PM}_{cf}=\frac{{PM}_{ref}}{{PM}_{raw}}$
eq 3	Barkjohn et al. (2021) US-wide correction	${PM}_{2.5}=0.524\times {PA}_{raw}-0.0862\;RH+5.75$
eq 4	Tryner et al. (2020), Charkrabarti et al. (2024)	${PM}_{2.5}=\frac{{PA}_{raw}}{1+\frac{0.25\;{RH}^{2}}{1-RH}}$

Two families of common, national corrections were tested with both PA_atm_ and PA_cf_1_, single versus mean channel measurements, and various sources of RH to find the combination with the highest accuracy. The two national corrections included the Barkjohn et al. (2021) US-wide correction and a nonlinear hygroscopic growth correction from Tryner et al. (2020) (eqs 3 and 4, Table 2).^7,13,26^ The PA data included single-channel measurements from both PA_atm_ and PA_cf_1_ and the mean of both channel a and b measurements from both PA_atm_ and PA_cf_1_. RH data was sourced from three places: uncorrected from the PA sensor itself (PARH), RH from the PA corrected by the Couzo et al. (2024) humidity correction (CRH), and RH locally obtained via the Northeast Regional Climate Center at the Philadelphia International Airport weather station (PHLRH).^21^ These combinations of local and national data corrections were selected with the goal of including the options that most researchers will have at their disposal when deploying PA for research (all detailed equations can be found in Supporting Information, Table SI1).

### 2.5 Evaluation Parameters

All data analyses were conducted in R Studio (R v.4.2.0). The following parameters were chosen to quantify the accuracy and precision. At the 1 h and 24 h levels, root-mean-square error (RMSE), mean bias error (MBE), linear regression slope coefficient (lm *b*), and the coefficient of determination (*R* ^2^) were calculated against the reference monitor. These four parameters were chosen to provide a comprehensive assessment of correction accuracy. RMSE was used to evaluate the influence of large deviations and the impact of extreme values on the performance. MBE was used to quantify the general systematic bias, indicating whether there is an over- or underestimation. Linear regression was used to assess how the correction performed across the range of observed values and to quantify the strength of the correction compared to the reference data. Pearson or Spearman correlation matrixes (Hmisc package) were used to quantify the non-zero precision of PA sensors at the hourly level in addition to the mean relative standard deviation (RSD) of all 1 h and all 24 h measurements.

## 3 Results and Discussion

### 3.1 PurpleAir Output Types and Relative Humidity Sources for Overall Performance

We found that averaging PA_atm_ channels a and b with uncorrected PARH was the best-performing combination of PA outputs and RH sources and that these outputs and RH were most accurate in the Barkjohn et al. (2021) US-wide model. Thus, BJ_atm_mean_PARH was the most accurate PM_2.5_ data correction for the combined pool of new and old sensors during the 2024 and 2025 Villanova roof collocations (Figure 1, Table 3, eq 5).

$$BJ\text{\_}atm\text{\_}mean\text{\_}PARH=0.524\times mean\;\left({pm}_{2.5}\text{\_}atm\text{\_}a,\;{pm}_{2.5{atm}_{b}}\right)-0.0862\times PARH+5.75$$ 5

**Figure 1:**
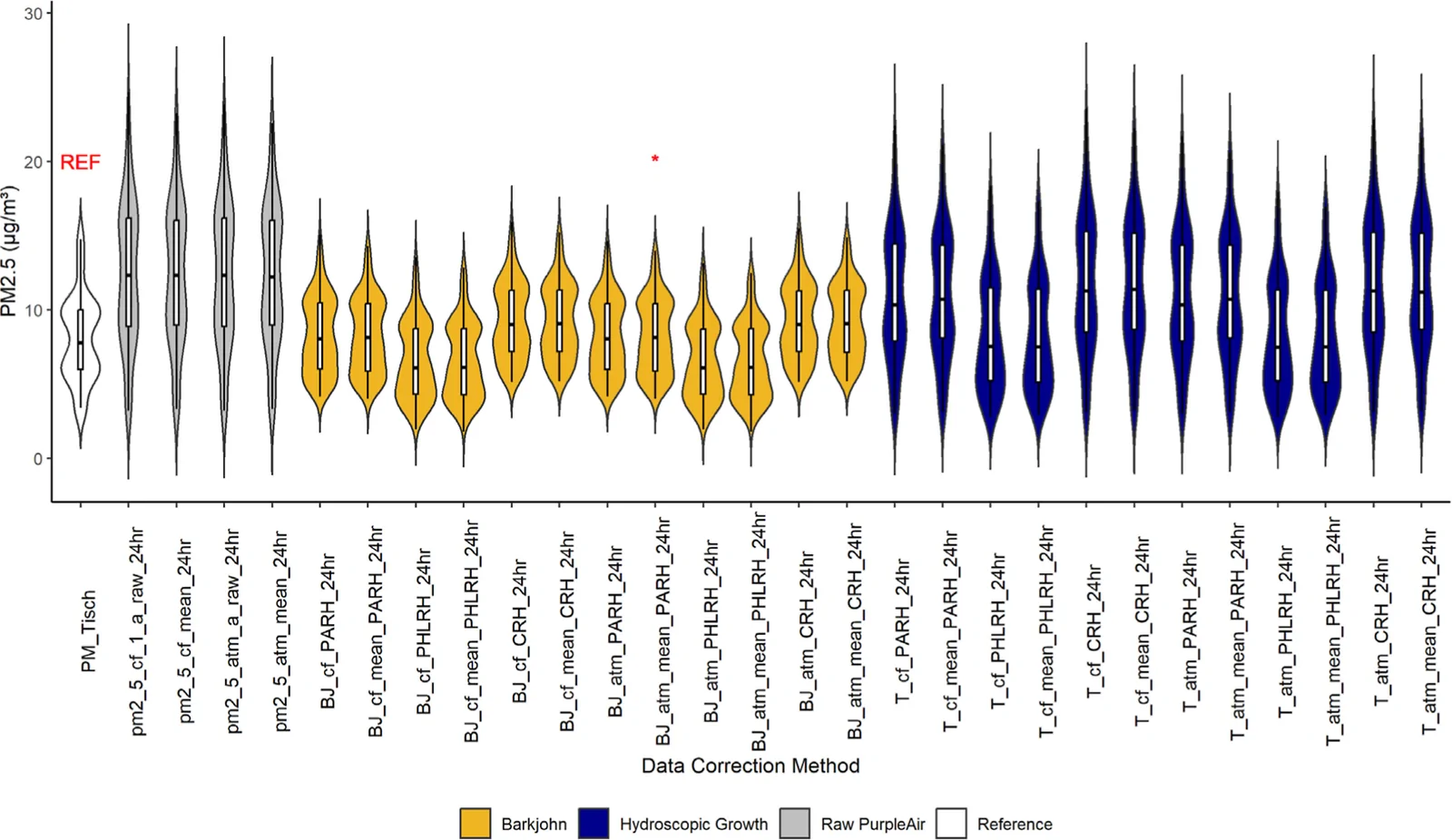
Agreement of average 24 h PM_2.5_ measurements of all PA sensors (*n* = 21) with data corrections and the Tisch Environmental gravimetric reference sampler (“REF”) from Villanova University collocation. Color represents different types of corrections. The * shows the correction with the highest overall accuracy. Violin plots show the probability density of the data, while the boxplot inside a violin shows the median value and interquartile range.

**Table 3:** Accuracy Results for All Data Corrections Analyzed from the PA Sensor Tested in Villanova, PA^a^

method	RMSE			MBE			lm *b*			lm R2		
	all	old	new	all	old	new	all	old	new	all	old	new
pm2_5_cf_1_a_raw_24 h	5.415	4.300	5.826	4.367	3.058	4.925	1.288	0.738	1.642	0.592	0.319	0.772
pm2_5_cf_mean_24 h	5.249	4.468	5.548	4.303	3.327	4.718	1.291	0.755	1.612	0.626	0.333	0.790
pm2_5_atm_a_raw_24 h	5.315	4.236	5.713	4.307	3.026	4.854	1.281	0.733	1.631	0.603	0.326	0.783
pm2_5_atm_mean_24 h	5.173	4.398	5.470	4.258	3.292	4.669	1.284	0.749	1.602	0.634	0.341	0.798
BJ_cf_PARH_24 h	1.769	2.261	1.503	0.161	–1.308	0.807	0.705	0.600	0.844	0.639	0.546	0.804
BJ_cf_mean_PARH_24 h	1.670	2.168	**1.395**	0.127	–1.167	0.697	0.707	0.609	0.829	0.673	0.555	0.820
BJ_cf_PHLRH_24 h	2.407	3.768	1.487	–1.537	–3.349	–0.765	0.689	0.708	0.805	0.602	0.612	0.797
BJ_cf_mean_PHLRH_24 h	2.356	3.632	1.514	–1.571	–3.207	–0.873	0.691	0.717	0.789	0.636	0.623	0.810
BJ_cf_CRH_24 h	2.106	1.949	2.172	1.151	–0.249	1.768	0.696	0.546	**0.848**	0.637	0.500	0.806
BJ_cf_mean_CRH_24 h	2.006	1.915	2.044	1.118	**–0.108**	1.657	0.698	0.555	0.832	0.672	0.511	0.824
BJ_atm_PARH_24 h	1.724	2.248	1.434	0.129	–1.324	0.768	0.701	0.597	0.839	0.653	0.558	0.820
**BJ_atm_mean_PARH_24 h**	**1.640**	2.153	1.354	**0.103**	–1.185	**0.670**	0.703	0.606	0.824	**0.683**	0.567	0.830
BJ_atm_PHLRH_24 h	2.396	3.767	1.462	–1.569	–3.365	–0.803	0.685	0.705	0.799	0.615	0.624	0.814
BJ_atm_mean_PHLRH_24 h	2.351	3.631	1.505	–1.595	–3.226	–0.899	0.687	0.714	0.784	0.645	0.635	0.820
BJ_atm_CRH_24 h	2.054	1.926	2.108	1.119	–0.266	1.729	0.693	0.544	0.842	0.651	0.511	0.821
BJ_atm_mean_CRH_24 h	1.969	**1.889**	2.004	1.094	–0.127	1.631	0.694	0.553	0.827	0.682	0.523	0.833
T_cf_PARH_24 h	3.943	3.051	4.277	2.878	1.540	3.466	1.199	0.802	1.492	0.637	0.418	0.807
T_cf_mean_PARH_24 h	3.779	3.159	4.022	2.824	1.783	3.282	1.203	0.820	1.465	0.672	0.432	0.827
T_cf_PHLRH_24 h	2.348	2.782	2.136	0.188	–1.829	1.048	1.016	1.004	1.192	0.612	0.630	0.782
T_cf_mean_PHLRH_24 h	2.188	2.664	1.950	0.145	–1.657	0.914	1.019	1.026	1.170	0.645	0.642	0.800
T_cf_CRH_24 h	4.706	3.681	5.093	3.658	2.346	4.235	1.240	0.762	1.566	0.610	0.361	0.787
T_cf_mean_CRH_24 h	4.541	3.824	4.822	3.598	2.603	4.036	1.244	0.779	1.538	0.645	0.376	0.806
T_atm_PARH_24 h	3.837	2.985	4.156	2.821	1.513	3.397	1.194	0.798	1.483	0.651	0.427	0.823
T_atm_mean_PARH_24 h	3.701	3.087	3.941	2.782	1.751	3.235	1.197	0.815	1.456	0.682	0.442	**0.836**
T_atm_PHLRH_24 h	2.265	2.759	2.017	0.143	–1.847	0.992	**1.013**	**1.001**	1.186	0.626	0.639	0.800
T_atm_mean_PHLRH_24 h	2.136	2.637	1.882	0.114	–1.677	0.878	1.015	1.023	1.164	0.654	**0.652**	0.809
T_atm_CRH_24 h	4.602	3.616	4.975	3.599	2.317	4.163	1.233	0.758	1.556	0.623	0.369	0.800
T_atm_mean_CRH_24 h	4.464	3.754	4.742	3.554	2.570	3.988	1.236	0.774	1.528	0.654	0.385	0.815

a New includes all new sensors from both 2024 and 2025 (*n* = 21). Old includes all sensors tested in 2025 after one year of field use (*n* = 5). Bolded values show the best results in each category.

BJ_atm_mean_PARH scored highest in three of the four categories tested for overall accuracy of new sensors and one-year-old sensors combined when compared to the gravimetric method (Table 3, RMSE = 1.640 μg/m^3^, MBE = 0.103 μg/m^3^, *R* ^2^ = 0.683). It is important to note that this data correction performed well across the three statistical metrics because each reflects different aspects of accuracy. The strong RMSE and MBE indicate that the magnitude of deviation is not frequently caused by extremes and that the systematic bias of the correction method is low. The moderate results from linear regression show that there is decent correlation with true PM_2.5_ values, but accuracy decreases at higher values of PM_2.5_. The decrease in accuracy at high values has been addressed previously with the development of data corrections for specific pollution events, like wildfires.^27^ No specific PM_2.5_ pollution events occurred during the Villanova collocations. The inclusion of all accuracy parameters in LCS air pollution studies is imperative for future comparisons of correction methods as they must be validated continuously.^28^

With BJ_atm_mean_PARH being the most accurate combination of output types, our results add a modification to Barkjohn et al. (2021) US-wide data correction. For the 24 h average, Barkjohn et al. (2021) found higher accuracy with PA_cf_1_ and evaluated their model with a single channel of data, rather than averaging channels a and b.^13^ Therefore, these results suggest modifying how the US-wide correction is implemented for research by using PA_atm_ and averaging both channels when using the PA PA-II flex air quality sensors.

Furthermore, with these inputs of the PA_atm_ mean of channels a and b with PARH (BJ_atm_mean_PARH), we found RMSE and MBE were lower by 0.9 μg/m^3^ and 0.4 μg/m^3^, respectively, than the reported results for the original Barkjohn US-wide correction in this region of the US (two study locations). This is contrary to the poor performance in accuracy found with nationwide data corrections, including Barkjohn et al. (2021), found in more extreme climates like the US South and Alaska, where localized corrections that considered both RH and temperature were determined to be more effective.^18,19^ We also found greater accuracy in the Tryner nonlinear hygroscopic growth correction with the newer model of PA than previously shown. When using the mean of PA_atm_ channels a and b along with PHLRH (T_atm_mean_PHLRH), the mean absolute error was 1.1 μg/m^3^ lower than corrected values found in Tryner et al. (2020) (Table 3, MBE = 1.114 μg/m^3^). This suggests that the corrections are becoming more effective as the models improve.

Among the three different RH sources tested (uncorrected RH from PA, corrected RH from PA, and RH from Philadelphia International Airport), uncorrected RH from the PA sensor itself resulted in the highest accuracy for the combination of all sensors (Table 3). Collectively, the data corrections implementing PARH had stronger linear regression results (average lm *b* = 0.10 higher, *R* ^2^ = 0.04 higher), whereas data corrections implementing PHLRH had a lower RMSE (average RMSE 0.452 μg/m^3^ lower). Therefore, we found little difference in PM_2.5_ accuracy whether RH data was obtained from a higher-grade weather station (PHLRH) or PA RH data (PARH). For overall performance, corrected RH from PA (CRH) performed well in the linear tests (average lm *b* = 0.966, *R* ^2^ = 0.65) but had high bias (average RMSE = 3.306 μg/m^3^, MBE = 2.361 μg/m^3^). With these differences, we determined that it is acceptable to source RH for data corrections from the uncorrected PARH.

### 3.2 Nationwide Data Corrections Outperforming Local Corrections

Of the two families of national data corrections, the Barkjohn US-wide group of corrected PM_2.5_ was more accurate, with an average RMSE of 2.03 μg/m^3^ and MBE of −0.106 μg/m^3^. These data corrections were most effective at capturing the range of true values of PM_2.5_ (Figure 1). The Tyrner family of nonlinear hygroscopic growth data corrections performed better linearly (average lm *b* = 1.15 and *R* ^2^ = 0.642), meaning that it could be more effective at higher ranges of PM_2.5_ (>15 μg/m^3^), but generally, it still overestimates true values of PM_2.5_. Uncorrected values of PA PM_2.5_ consistently scored the lowest accuracy and produced the widest range of values due to higher overestimation (Figure 1).

In addition to outperforming the other nationwide corrections, BJ_atm_mean_PARH also performed better than the two locally developed data corrections during the two collocation periods in West Philadelphia. In this urban environment, BJ_atm_mean_PARH was more accurate than the local linear correction developed by comparison with the gravimetric method in VU collocations (Gravimetric_cf), the local linear correction developed with comparison to the nearest government monitoring station (Norristown_cf), and the raw PA PM_2.5_ (pm2_5_atm_mean; all equations found in Table SI2a). Specifically, in West Philadelphia, based on collocated measurements with gravimetric measurements conducted in Summer 2024 and Spring 2025, BJ_atm_mean_PARH had the highest accuracy in all four categories (Figure 2 and Table SI2b, RMSE = 1.605 μg/m^3^, MBE = 0.521 μg/m^3^, lm *b* = 0.950, *R* ^2^ = 0.841). The BJ_atm_mean_PARH was most successful at limiting the overestimation of true PM_2.5_, whereas the local linear corrections, Gravimetric_cf and Norristown_cf, showed poor accuracy by failing to lower PM_2.5_ values at higher concentrations (Figure 2 and Table SI2b, RMSE = 2.350 μg/m^3^ and 2.48 μg/m^3^, respectively). Bias in the raw PM_2.5_ was much higher, with RMSE scores of 6.00 μg/m^3^ in West Philadelphia (Figure 2 and Table SI2b). With accuracy this low, we do not recommend using uncorrected PA PM_2.5_ values for research purposes.

**Figure 2:**
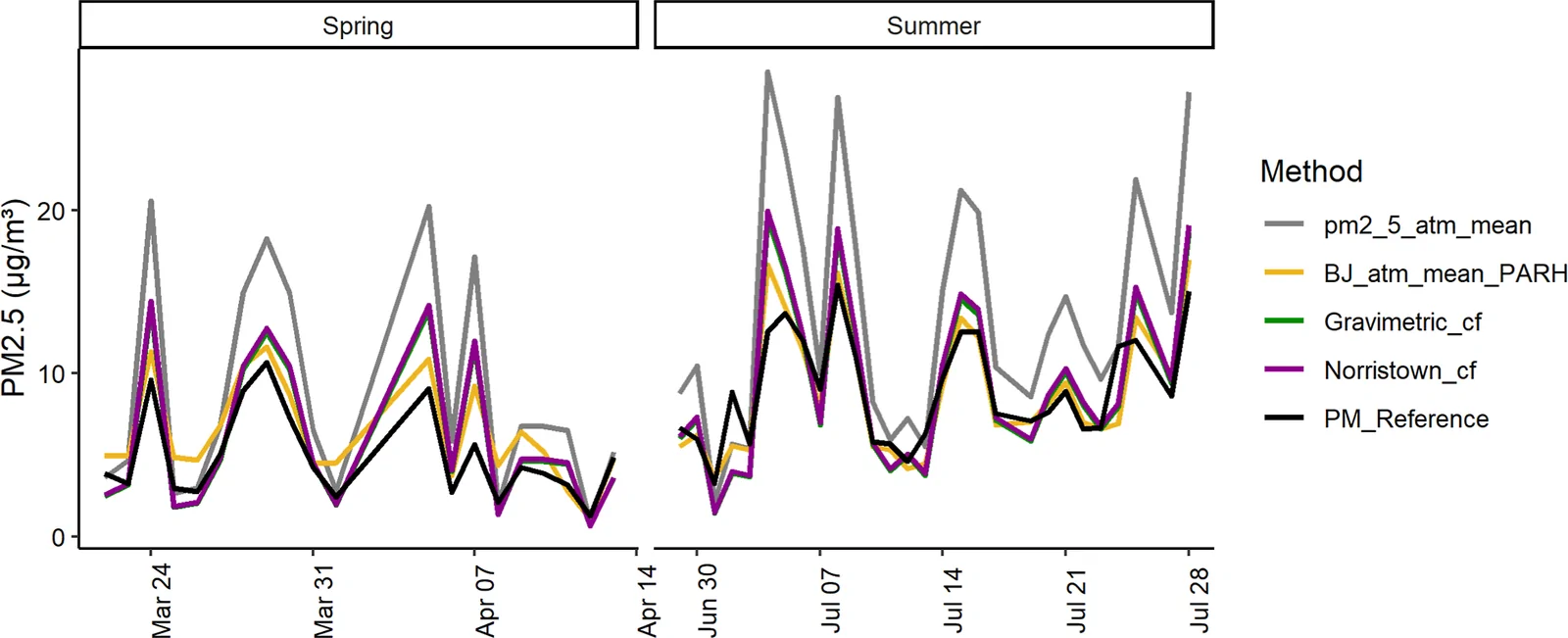
PA PM_2.5_ data corrections including raw atm outputs, a modified US-wide correction, and two local linear corrections, compared against a gravimetric reference monitor for a 24 h measurement in West Philadelphia.

Additionally, the BJ_atm_mean_PARH performed better than other data corrections in the literature that have been previously used for PA sensors. In the same geographic region of this study, BJ_atm_mean_PARH scored a mean absolute error 2.6 μg/m^3^ lower than a previous hygroscopic growth correction developed in Pittsburgh.^14^ Furthermore, we found higher accuracy by RMSE and MBE here with the modified US-wide correction, BJ_atm_mean_PARH, compared to both the US-wide correction and a Baltimore area specific correction found by a Maryland-based study.^16^ Both these studies deployed PA sensors equipped with PMS-5003 particle counters. The differences could be attributed to the use of the newer PA-II flex air quality sensor with PMS-6003 used in this study.

Another explanation for BJ_atm_mean_PARH’s high accuracy would be the weather in the Northeast US region. PA sensors seem to perform better in regions that do not frequently experience extreme weather. Temperature and humidity play great roles in affecting accuracy. For instance, in an urban study in Greece, PA accuracy decreased as RH increased when compared to the reference monitor (RH range: 30–90%).^9^ Additionally, in the US South, the constant high humidity caused other local data corrections to be more effective than the Barkjohn model (annual humidity ranges: 65.5–74.0%).^18^ At the daily scale, BJ_atm_mean_PARH had lower RMSE and higher *R* ^2^ than the new data correction that was developed by Mathieu-Campbell and others (2024) for the high humidity in the US South. Extreme temperatures also have an effect on PA performance, explaining why no previous data corrections were proven to be suitable in Alaska (temperature range = −42.1–14.8 °C).^19^ In this study, the collocation sites had moderate weather (average weather across all collocations: daily temperature = 22.5 °C, daily RH = 67.0%, precipitation on 41% of days). These factors could be the reasons for the high accuracy of the data corrections found in this study of the Mid-Atlantic region. There were no extreme temperatures, and there was consistently moderate humidity. Furthermore, precipitation can also lower ambient PM_2.5_, and PA sensors are more effective at lower ranges.^29^

Our study from the urban environment in the Mid-Atlantic region shows high trustworthiness in the PA PM_2.5_ data. While these findings may be broadly applicable to other temperate urban environments, further validation in other temperate regions is needed. In moderate climates, unlike what was found by Leenose and others (2025) and Mathieu-Campbell and others (2024) in the extreme cold and extreme heat, respectively, the nationwide data corrections are suitable. Our data support the use of the US-wide PM_2.5_ correction, rather than a new local correction. We speculate that the climate is the driving reason behind this, acknowledging that research in extreme climates would still likely find a local correction to be more accurate. With this, it will be most important for future applications of PA to consider their temperature range during field deployment when choosing a correction equation. Recalibration should also be considered when sensors are deployed in environments with substantial changes in the ambient temperature. In moderate climate regions, we can recommend implementing our methodology here, as it leads to higher accuracy than previously shown, and the region is well-suited for PA sensors. The findings of this study are influenced by both climatic conditions and deployment characteristics, which may limit their generalization to other environments.

### 3.3 1 h versus 24 h Measurements

The BJ_atm_mean_PARH correction was most accurate at measuring PM_2.5_ on the 1 h time scale. In comparison to Teledyne T640 in the Northeast Philadelphia collocation, this correction method resulted in an RMSE of 3.096 μg/m^3^, an MBE of −0.108 μg/m^3^, a lm *b* of 0.762, and an *R* ^2^ of 0.753 (Figure 3 and Table SI3). It is important to note that Northeast Philadelphia collocation was the only scenario in which the systematic bias underestimated true values of PM_2.5_ after the BJ_atm_mean_PARH was applied to a new sensor. The higher RMSE was likely caused by the extreme spike in pollution seen on July 4th’s Independence Day firework celebrations. This was confirmed through sensitivity analysis, as RMSE, MBE, and lm *b* improved after removing those values (Table SI3). Therefore, BJ_atm_mean_PARH was successful at measuring PM_2.5_ at both the 1 h and 24 h time scales in Philadelphia (Figures 2 and 3). Previous findings about PA measurements on different time scales are less clear. For instance, during wildfire events in California, PA sensors were found to be more accurate on the 10 min interval than the 3 h interval.^30^ Even though it is common practice to apply data cleaning parameters for PA PM_2.5_ validation, the 24 h scale further minimizes error in sensor performance and was found to be more accurate than the 1 h scale.

**Figure 3:**
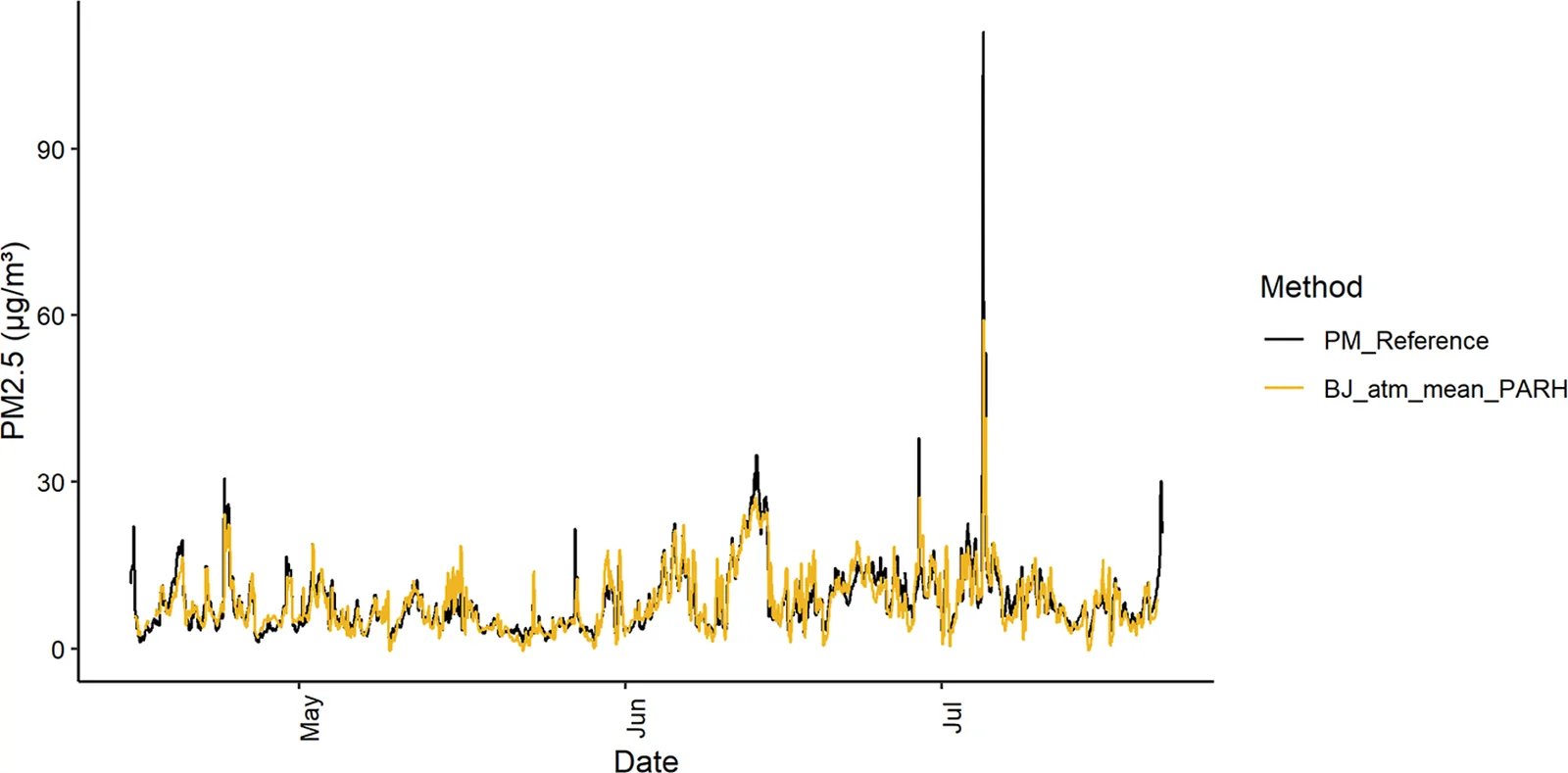
PA PM_2.5_ corrected by the modified US-wide correction compared against Teledyne T640 continuous reference monitor for hourly measurement in Northeast Philadelphia.

### 3.4 Accuracy Change after 1 Year of Field Deployment

When comparing new and one-year-old PA sensors, we found accuracy decreased for both raw PA PM_2.5_ and corrected PM_2.5_ after the sensors were deployed in the field for one year (duration of continuous field deployment was 11.5 months, Figure 4). The most accurate overall data correction method, BJ_atm_mean_PARH, increased by 0.799 μg/m^3^ RMSE and decreased by 1.288 μg/m^3^ MBE, with the correction now producing an underestimation bias (Table 3). Previous work has shown that 2 year-old sensors have an increase in bias, not an underestimation.^31^ The change in accuracy is typically due to sensor drift over time, or frequent exposure to elevated levels of PM_2.5_. The underestimation seen here is a deviation from the raw PA PM_2.5_, which consistently overestimates true values.^11,28^ The BJ_atm_mean_PARH correction also produced poor linear regression results lm *b* and *R* ^2^ (Table 3, lm *b* = 0.606, *R* ^2^ = 0.567). These results are far from the expected marks for good accuracy, which would be lm *b* and *R* ^2^ closer to 1. Recommending a single correction approach for one-year-old sensors is challenging, as none of the nationwide correction methods performed consistently across all four accuracy metrics (Table 3). These findings suggest that newer models of PA also show possible performance degradation after one year of deployment based on a subset of five sensors tested over a short period of time (for 11 days during summer). For example, *R* ^2^ values ranged from 0.54 to 0.86 compared to government reference monitors when testing previous models over a seven year period in California.^22^ The decrease in accuracy after one-year of field use shows that it is especially important to apply a data correction method for older PA sensors for long-term studies. Sensor drift can occur in extreme environmental conditions and may require a different model for improved performance as shown in a study with high humidity conditions in the southeastern United States.^18^ However, depending on the region’s climate, PA sensor performance may not degrade in the region without major seasonal shifts or significant temperature shocks. Several studies have indicated that ambient temperature and relative humidity are the main predictor variables affecting PA sensor performance.^13,18^

**Figure 4:**
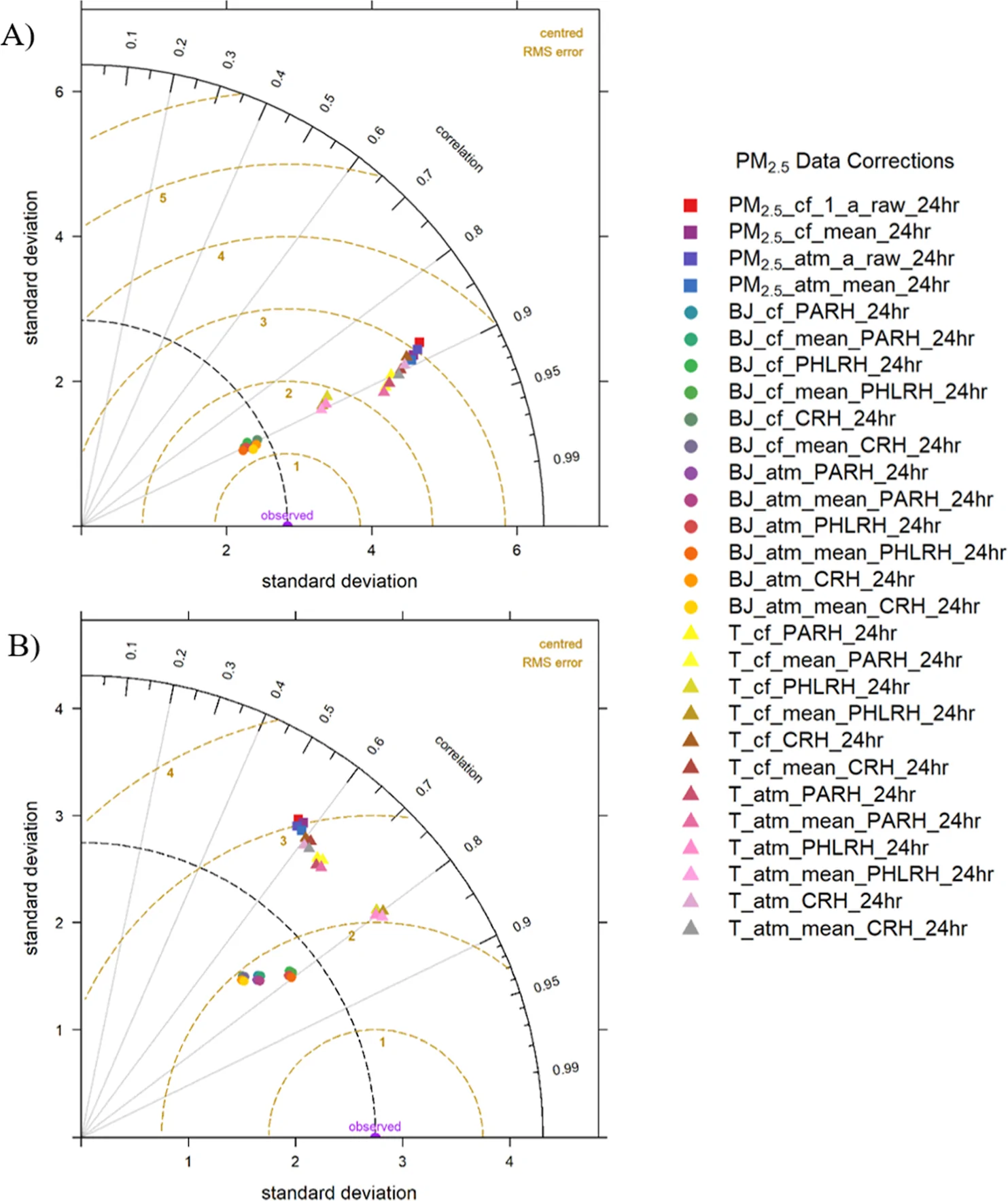
Taylor diagrams showing accuracy results for (A) new PA sensors (*n* = 21) and for (B) 1 year-old PA sensors (*n* = 5) from collocation experiments in Villanova, Pennsylvania. Observed values are 24 h gravimetric PM_2.5_ measurements using a Tisch low-volume PM sampler.

Combining the Barkjohn US-wide PM_2.5_ correction with the Couzo et al. (2024) PA RH correction method may enhance the longevity of accurate PA sensor measurements. In one-year-old sensors, we found that RMSE was reduced by 1.04 μg/m^3^ and the MBE by 2.08 μg/m^3^ when using the Barkjohn US-wide PM_2.5_ correction with CHR instead of using PARH or PHLRH (Table 3, all equations with “BJ” and “CRH”). Couzo et al. found that PA sensors underestimate ambient RH by 17.4%.^21^ It is plausible that as the sensor’s RH data drifts over time, combining both the PA RH correction and the Barkjohn et al. (2021) US-wide PM_2.5_ correction provides a viable method for extending the lifespan of accurate PA sensor measurements. Further work should confirm if this result is seen when sensors age past one year. A longitudinal drift analysis would also be valuable. However, such analysis was not feasible in the present study because the same sensors were not continuously collocated with reference monitors throughout the deployment period.

### 3.5 Precision before and after 1 Year of Field Deployment

We found that both new and 1 year-old PA sensors have high precision (Figure 5, new *n* = 16, old *n* = 5). With an increase in sensor bias seen with PA sensors, we expected to also see a decrease in precision. However, we saw little degradation in non-zero precision between PA sensors after 1 year of continuous field use (Figure 5). Mean correlation R values increased from 0.978 to 0.995 when comparing new out-of-the-box sensors to old sensors. Median RSD for new sensors measuring at 24 h was found to be 2.21%, while 1 year-old sensors increased to 6.87% (Table SI4). With respect to measuring at different time scales, 24 h measurements had higher precision than 1 h measurements. Corrected 24 h PM_2.5_ had a lower median RSD (2.21%) than corrected 1 h PM_2.5_ (RSD = 2.75%) for new sensors (Table SI4). This is consistent with previous work in that sensors before and after deployment have similar RSD precision, but different in that RSD values were lower overall than the older models (RSD less than 20% at PM_2.5_ values ≥9 μg/m^3^).^7^ These results lead to a high confidence in comparing PM_2.5_ from different sensors of the newer models deployed in the field.

**Figure 5:**
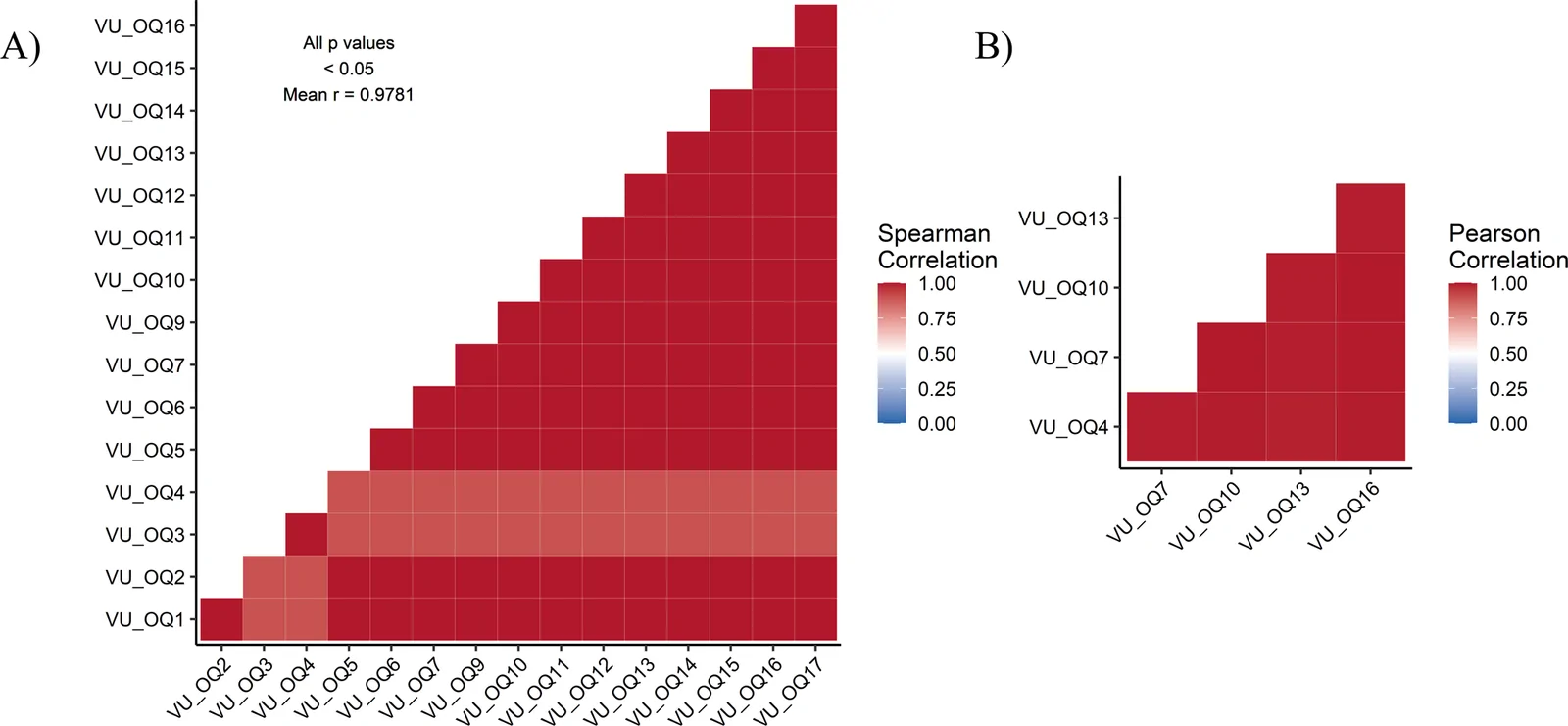
Correlation matrixes of (A) new PA sensors from 2024 (*n* = 16) and (B) one-year-old PA sensors from 2025 (*n* = 5) with the BJ_atm_mean_PARH data correction applied. Correlation coefficients and *p* values are displayed on the graphs.

### 3.6 Limitations

Evaluation of PA sensors was conducted only in summer and spring seasons. The effect of cold temperatures during the winter season in the Mid-Atlantic on the sensor performance was not evaluated. It is possible that sensor drift after one year of deployment occurred due to low temperatures during winter seasons and that sensor performance may not vary over longer deployments in regions without extreme cold temperatures, such as stable tropical environments. Therefore, the results of PA sensor evaluation in this study are region-specific and are primarily applicable to the Mid-Atlantic region, particularly Pennsylvania. The results of sensor performance after one year of deployment are based on only a subset of five sensors out of a total of 15 sensors deployed during the study. Only an 11 day collocation was conducted during summer to test a subset of five sensors after one year of deployment. Therefore, the limited temporal scope of testing contributes to a major source of uncertainty in conclusions about sensor performance after one year of deployment. Finally, it is also limited by the 24 h and 1 h comparisons. Since PA reports on 2 min intervals, this testing should be repeated with a reference monitor that can mimic the 2 or 10 min time scale to test all measurement scales.

### 3.7 Summary and Recommendations

This study shows that the Barkjohn et al. (2021) US-wide correction for PA sensors with slight modification (BJ_atm_mean_PARH) to use PA_atm_ and mean of both channels provides effective and reasonably accurate PM_2.5_ data (Figure 1, Table 3, RMSE = 1.640 μg/m^3^, MBE = 0.103 μg/m^3^, lm *b* = 0.703, *R* ^2^ = 0.683, new sensor non-zero precision: RSD = 2.21%). In this study, the evaluation was conducted by comparing the simplified local correction method with established nationwide calibration methods. In the moderate climate of Pennsylvania, United States, the nationwide PM_2.5_ correction was more adequate than a locally derived correction. This is opposite to what has been found in more extreme climates with higher and lower temperatures. Additionally, we found that accuracy degrades after 1 year of field use, while precision between sensors only slightly degrades when applying BJ_atm_mean_PARH (Figure 4, 1 year-old accuracy: RMSE = 2.153 μg/m^3^, MBE = −1.185 μg/m^3^, lm *b* = 0.606, *R* ^2^ = 0.567, 1 year-old 24 h precision: RSD = 6.87%). Future work should consider how data corrections might change as sensors age to prolong the lifespan of the LCS. Here, we found that correcting PARH with the Couzo et al. (2024) RH correction to input into the US-wide correction is one avenue for that possibility. Producing new data corrections for older sensors with machine learning would be a strong approach to further test this, as machine learning correction models have been shown to increase PA accuracy.^32,33^ In conclusion, this study further shows why regional validation is imperative for low-cost air quality monitoring as performance can greatly vary.

## Supplementary Material



## Notes

Data correction equation tables and accuracy and precision result tables (PDF)

## References

[1] Sokhi R. S., Moussiopoulos N., Baklanov A., Bartzis J., Coll I., Finardi S., Friedrich R., Geels C., Grönholm T., Halenka T., Ketzel M., Maragkidou A., Matthias V., Moldanova J., Ntziachristos L., Schäfer K., Suppan P., Tsegas G., Carmichael G., Franco V., Hanna S., Jalkanen J.-P., Velders G. J. M., Kukkonen J. (2022). Advances in Air Quality Research – Current and
Emerging Challenges. Atmos. Chem. Phys..

[2] Karagulian F., Barbiere M., Kotsev A., Spinelle L., Gerboles M., Lagler F., Redon N., Crunaire S., Borowiak A. (2019). Review of
the Performance of Low-Cost Sensors for Air Quality Monitoring. Atmosphere.

[3] Castell N., Dauge F. R., Schneider P., Vogt M., Lerner U., Fishbain B., Broday D., Bartonova A. (2017). Can Commercial
Low-Cost Sensor Platforms Contribute to Air Quality Monitoring and
Exposure Estimates?. Environ. Int..

[4] Gardner-Frolick R., Boyd D., Giang A. (2022). Selecting Data Analytic
and Modeling
Methods to Support Air Pollution and Environmental Justice Investigations:
A Critical Review and Guidance Framework. Environ.
Sci. Technol..

[5] PurpleAir Real-time Air Quality Monitoring by PurpleAir. https://www2.purpleair.com/ . 2024. (accessed October 10, 2024).

[6] PurpleAir Community What is the Difference between CF = 1, ATM, and ALT? - Data/API. 2023. https://community.purpleair.com/t/what-is-the-difference-between-cf-1-atm-and-alt/6442 (accessed October 07, 2024).

[7] Tryner J., L’Orange C., Mehaffy J., Miller-Lionberg D., Hofstetter J. C., Wilson A., Volckens J. (2020). Laboratory Evaluation
of Low-Cost PurpleAir PM Monitors and in-Field Correction Using Co-Located
Portable Filter Samplers. Atmos. Environ..

[8] Heintzelman A., Filippelli G. M., Moreno-Madriñan M. J., Wilson J. S., Wang L., Druschel G. K., Lulla V. O. (2023). Efficacy of Low-Cost
Sensor Networks at Detecting Fine-Scale Variations in Particulate
Matter in Urban Environments. Int. J. Environ.
Res. Public Health.

[9] Stavroulas I., Grivas G., Michalopoulos P., Liakakou E., Bougiatioti A., Kalkavouras P., Fameli K. M., Hatzianastassiou N., Mihalopoulos N., Gerasopoulos E. (2020). Field Evaluation of Low-Cost PM Sensors
(Purple Air PA-II) Under Variable Urban Air Quality Conditions, in
Greece. Atmosphere.

[10] Wallace L., Bi J., Ott W. R., Sarnat J., Liu Y. (2021). Calibration of Low-Cost
PurpleAir Outdoor Monitors Using an Improved Method of Calculating
PM2.5. Atmos. Environ..

[11] Gualtieri G., Brilli L., Carotenuto F., Cavaliere A., Giordano T., Putzolu S., Vagnoli C., Zaldei A., Gioli B. (2024). Performance Assessment of Two Low-Cost PM2.5 and PM10 Monitoring
Networks in the Padana Plain (Italy). Sensors.

[12] Molina
Rueda E., Carter E., L’Orange C., Quinn C., Volckens J. (2023). Size-Resolved Field Performance of
Low-Cost Sensors for Particulate Matter Air Pollution. Environ. Sci. Technol. Lett..

[13] Barkjohn K. K., Gantt B., Clements A. L. (2021). Development and
Application of a
United States-Wide Correction for PM_2.5_ Data Collected
with the PurpleAir Sensor. Atmos. Meas. Tech..

[14] Malings C., Tanzer R., Hauryliuk A., Saha P. K., Robinson A. L., Presto A. A., Subramanian R. (2020). Fine Particle
Mass Monitoring with
Low-Cost Sensors: Corrections and Long-Term Performance Evaluation. Aerosol Sci. Technol..

[15] Psotka J., Tracey E., Sica R. J. (2025). Retrieval of Bulk
Hygroscopicity
from PurpleAir PM_2.5_ Sensor Measurements. Atmos. Meas. Tech..

[16] Koehler K., Wilks M., Green T., Rule A. M., Zamora M. L., Buehler C., Datta A., Gentner D. R., Putcha N., Hansel N. N., Kirk G. D., Raju S., McCormack M. (2023). Evaluation
of Calibration Approaches for Indoor Deployments of PurpleAir Monitors. Atmos. Environ..

[17] Ardon-Dryer K., Dryer Y., Williams J. N., Moghimi N. (2020). Measurements of PM_2.5_ with PurpleAir under
Atmospheric Conditions. Atmos. Meas. Tech..

[18] Mathieu-Campbell M. E., Guo C., Grieshop A. P., Richmond-Bryant J. (2024). Calibration of PurpleAir Low-Cost
Particulate Matter Sensors: Model Development for Air Quality under
High Relative Humidity Conditions. Atmos. Meas.
Tech..

[19] Leenose J. F., Vilagi A., Pride D., Betha R., Aggarwal S. (2025). Unveiling
the Limits of Existing Correction Factors for a Low-Cost PM2.5 Sensor
in Cold Environments and Development of a Tailored Solution. ACS ES&T Air.

[20] PurpleAir Community PurpleAir Sensors Functional Overview - Sensors. 2021. https://community.purpleair.com/t/purpleair-sensors-functional-overview/150 (accessed October 10, 2024).

[21] Couzo E., Valencia A., Gittis P. (2024). Evaluation and Correction of PurpleAir
Temperature and Relative Humidity Measurements. Atmosphere.

[22] Farooqui Z., Biswas J., Saha J. (2023). Long-Term
Assessment of PurpleAir
Low-Cost Sensor for PM2.5 in California, USA. Pollutants.

[23] Sylvester C., Strader S., Armstead J., Shabazz J., Shakya K. M. (2026). Characterization
of Indoor and Outdoor Urban Particle and Gas Pollution at Neighborhood
Scale in Philadelphia, Pennsylvania. Environ.
Pollut..

[24] City of Philadelphia Air Management Services . 2023-2024 Air Monitoring Network Plan, 2023. https://www.phila.gov/documents/air-management-reports-and-documents/ (accessed July 10, 2025).

[25] National Oceanic and Atmospheric Administration . Geographical Reference Maps | U.S. Climate Regions | National Centers for Environmental Information (NCEI). https://www.ncei.noaa.gov/access/monitoring/reference-maps/us-climate-regions. 2025. (accessed September 22, 2025).

[26] Chakrabarti B., Fine P. M., Delfino R., Sioutas C. (2004). Performance Evaluation
of the Active-Flow Personal DataRAM PM2.5 Mass Monitor (Thermo Anderson
pDR-1200) Designed for Continuous Personal Exposure Measurements. Atmos. Environ..

[27] Barkjohn K. K., Holder A. L., Frederick S. G., Clements A. L. (2022). Correction and Accuracy
of PurpleAir PM2.5 Measurements for Extreme Wildfire Smoke. Sensors.

[28] Zimmerman N. (2022). Tutorial:
Guidelines for Implementing Low-Cost Sensor Networks for Aerosol Monitoring. J. Aerosol Sci..

[29] Sun Y., Zhao C., Su Y., Ma Z., Li J., Letu H., Yang Y., Fan H. (2019). Distinct Impacts
of
Light and Heavy Precipitation on PM2.5 Mass Concentration in Beijing. Earth Space Sci..

[30] Stampfer O., Zuidema C., Allen R. W., Fox J., Sampson P., Seto E., Karr C. J. (2024). Practical Considerations for Using
Low-Cost Sensors to Assess Wildfire Smoke Exposure in School and Childcare
Settings. J. Exposure Sci. Environ. Epidemiol..

[31] Bi J., Wildani A., Chang H. H., Liu Y. (2020). Incorporating Low-Cost
Sensor Measurements into High-Resolution PM_2.5_ Modeling
at a Large Spatial Scale. Environ. Sci. Technol..

[32] Raheja G., Nimo J., Appoh E. K.-E., Essien B., Sunu M., Nyante J., Amegah M., Quansah R., Arku R. E., Penn S. L., Giordano M. R., Zheng Z., Jack D., Chillrud S., Amegah K., Subramanian R., Pinder R., Appah-Sampong E., Tetteh E. N., Borketey M. A., Hughes A. F., Westervelt D. M. (2023). Low-Cost
Sensor Performance Intercomparison,
Correction Factor Development, and 2+ Years of Ambient PM2.5 Monitoring
in Accra, Ghana. Environ. Sci. Technol..

[33] Ghahremanloo M., Choi Y., Momeni M. (2025). Deep Learning
Calibration Model for
PurpleAir PM2.5 Measurements: Comprehensive Investigation of the PurpleAir
Network. Atmos. Environ..

