# Supplementary material for: Validation of PurpleAir PM2.5 Sensors in Temperate Urban Environments
Source: ACS EST Air. 2026 Sep 18;3(10):2503–12. doi: 10.1021/acsestair.5c00443 (PMC13660415; doi:10.1021/acsestair.5c00443)
Supplement: Supplementary file 1 [file ea5c00443_si_001.pdf]

Supplementary Information:

Validation of PurpleAir $PM_{2.5}$ Sensors in Temperate Urban Environments

Charles C. Sylvester,[1] Nathaniel D. Hauenstein,[1] Kabindra M. Shakya[1*]

[1] Villanova University

[*] Corresponding author, kabindra.shakya@villanova.edu

List of Tables:

Table SI 1: Data correction equation table for Villanova Collocations.

Table SI 2: (a) Data correction equation table and (b) accuracy results table for West Philadelphia Collocations.

Table SI 3: Accuracy results table for Northeast Philadelphia collocation with and without July 4th spike in $PM_{2.5}$.

Table SI 4: Non-zero precision results table.

SI 1 Equations to formulate each data correction model used during the Villanova collocations with PurpleAir sensors. PA_RH is RH measured from corresponding PurpleAir sensors, and PHL_RH is the RH data obtained from the weather station at Philadelphia International Airport.

Method	Equation
pm2_5_cf_1_a_raw_24hr	pm2_5_cf_1_a
pm2_5_cf_mean_24hr	mean(pm2_5_cf_1_a, pm2_5_cf_1_b)
pm2_5_atm_a_raw_24hr	pm2_5_atm_a
pm2_5_atm_mean_24hr	mean(pm2_5_atm_a, pm2_5_atm_b)
BJ_cf_PARH_24hr	0.524 * pm2_5_cf_1_a - 0.0862 * PA_RH + 5.75
BJ_cf_mean_PARH_24hr	0.524 * mean(pm2_5_cf_1_a, pm2_5_cf_1_b) - 0.0862 * PA_RH + 5.75
BJ_cf_PHLRH_24hr	0.524 * pm2_5_cf_1_a - 0.0862 * PHL_RH + 5.75
BJ_cf_mean_PHLRH_24hr	0.524 * mean(pm2_5_cf_1_a, pm2_5_cf_1_b) - 0.0862 * PHL_RH + 5.75
BJ_cf_CRH_24hr	0.524 * pm2_5_cf_1_a - 0.0862 * (0.75 * PA_RH + 0.12) + 5.75
BJ_cf_mean_CRH_24hr	0.524 * mean(pm2_5_cf_1_a, pm2_5_cf_1_b) - 0.0862 * (0.75 * PA_RH + 0.12) + 5.75
BJ_atm_PARH_24hr	0.524 * pm2_5_atm_a - 0.0862 * PA_RH + 5.75
BJ_atm_mean_PARH_24hr	0.524 * mean(pm2_5_atm_a, pm2_5_atm_b) - 0.0862 * PA_RH + 5.75
BJ_atm_PHLRH_24hr	0.524 * pm2_5_atm_a - 0.0862 * PHL_RH + 5.75
BJ_atm_mean_PHLRH_24hr	0.524 * mean(pm2_5_atm_a, pm2_5_atm_b) - 0.0862 * PHL_RH + 5.75
BJ_atm_CRH_24hr	0.524 * pm2_5_atm_a - 0.0862 * (0.75 * PA_RH + 0.12) + 5.75
BJ_atm_mean_CRH_24hr	0.524 * mean(pm2_5_atm_a, pm2_5_atm_b) - 0.0862 * (0.75 * PA_RH + 0.12) + 5.75
T_cf_PARH_24hr	pm2_5_cf_1_a / [1 + (0.25 * (PA_RH/100)^2)/(1 - (PA_RH/100))]
T_cf_mean_PARH_24hr	mean(pm2_5_cf_1_a, pm2_5_cf_1_b) / [1 + (0.25 * (PA_RH/100)^2)/(1 - (PA_RH/100))]
T_cf_PHLRH_24hr	pm2_5_cf_1_a / [1 + (0.25 * (PHL_RH/100)^2)/(1 - (PHL_RH/100))]
T_cf_mean_PHLRH_24hr	mean(pm2_5_cf_1_a, pm2_5_cf_1_b) / [1 + (0.25 * (PHL_RH/100)^2)/(1 - (PHL_RH/100))]
T_cf_CRH_24hr	pm2_5_cf_1_a / [1 + (0.25 * ((0.75 * PA_RH + 0.12)/100)^2)/(1 - ((0.75 * PA_RH + 0.12)/100))]
T_cf_mean_CRH_24hr	mean(pm2_5_cf_1_a, pm2_5_cf_1_b) / [1 + (0.25 * ((0.75 * PA_RH + 0.12)/100)^2)/(1 - ((0.75 * PA_RH + 0.12)/100))]
T_atm_PARH_24hr	pm2_5_atm_a/ [1 + (0.25 * (PA_RH/100)^2)/(1 - (PA_RH/100))]
T_atm_mean_PARH_24hr	mean(pm2_5_atm_a, pm2_5_atm_b) / [1 + (0.25 * (PA_RH/100)^2)/(1 - (PA_RH/100))]
T_atm_PHLRH_24hr	pm2_5_atm_a / [1 + (0.25 * (PHL_RH/100)^2)/(1 - (PHL_RH/100))]
T_atm_mean_PHLRH_24hr	mean(pm2_5_atm_a, pm2_5_atm_b) / [1 + (0.25 * (PHL_RH/100)^2)/(1 - (PHL_RH/100))]
T_atm_CRH_24hr	pm2_5_atm_a / [1 + (0.25 * ((0.75 * PA_RH + 0.12)/100)^2)/(1 - ((0.75 * PA_RH + 0.12)/100))]
T_atm_mean_CRH_24hr	mean(pm2_5_atm_a, pm2_5_atm_b) / [1 + (0.25 * ((0.75 * PA_RH + 0.12)/100)^2)/(1 - ((0.75 * PA_RH + 0.12)/100))]

SI 2a Equations for data corrections in West Philadelphia PurpleAir collocation with Tish Environmental gravimetric reference sampler. Gravimetric_cf and Norristown_cf we generated during Villanova University collocation and applied during the West Philadelphia collocation. Results correspond with Figure 2.

Method	Equation
BJ_atm_mean_PARH	0.524 * mean(pm2_5_atm_a, pm2_5_atm_b) - 0.0862 * PA_RH + 5.75
Gravimetric_cf	0.684 * mean(pm2_5_atm_a, pm2_5_atm_b)
Norristown_cf	0.700 * mean(pm2_5_atm_a, pm2_5_atm_b)
pm2_5_atm_mean	mean(pm2_5_atm_a, pm2_5_atm_b)

SI 2b Accuracy results from PurpleAir PurpleAir $PM_{2.5}$ data corrections including raw atm outputs, modified US-wide correction, and two local linear corrections compared against gravimetric reference monitor for 24-hour measurement in West Philadelphia. Results correspond with Figure 2. Ranges in parentheses represent bootstrapped 95% confidence intervals using 5000 bootstrap resamples.

Method	RMSE (μg/m^3)	MBE (μg/m^3)	lm b	R2
BJ_atm_mean_PARH	1.60543 (1.276, 2.094)	0.520513 (0.0640, 0.9153)	0.949606 (0.8030, 1.0649)	0.840767 (0.6719, 0.9150)
Gravimetric_cf	2.350355 (1.852, 3.060)	0.560703 (-0.0568, 1.2126)	1.248561 (1.098, 1.397)	0.825422 (0.6644, 0.9091)
Norristown_cf	2.482259 (1.959, 3.225)	0.740917 (0.1049, 1.4273)	1.277125 (1.124, 1.429)	0.825422 (0.6644, 0.9091)
pm2_5_atm_mean	5.999714 (4.850, 7.373)	4.193507 (3.036, 5.442)	1.824364 (1.605, 2.042)	0.825422 (0.6644, 0.9091)

SI 3 Accuracy results for Raw PurpleAir $PM_{2.5}$ and PurpleAir $PM_{2.5}$ corrected by the modified US-wide correction compared against Teledyne T640 continuous reference monitor for hourly measurement in Northeast Philadelphia. Results correspond with Figure 3. Ranges in parentheses represent bootstrapped 95% confidence intervals using 5000 bootstrap resamples.

Method	RMSE (μg/m^3)	MBE (μg/m^3)	lm b	R2
BJ_atm_mean_PARH	3.09647 (2.430, 4.649)	-0.10822 (-0.2572, 0.0025)	0.762054 (0.5870, 0.8761)	0.753034 (0.6056, 0.8152)
pm2_5_atm_mean	7.77289 (7.451, 8.211)	4.607994 (4.367, 4.881)	1.41588 (1.083, 1.621)	0.705211 (0.5659, 0.7649)
Excluding July 4th spike				
BJ_atm_mean_PARH	2.902 (2.341, 4.703)	-0.0842 (-0.2223, 0.0226)	0.792 (0.5576, 0.8887)	0.747 (0.5777, 0.8162)
pm2_5_atm_mean	7.47 (7.183, 7.872)	4.46 (4.229, 4.722)	1.451 (0.983, 1.655)	0.662 (0.5040, 0.7358)

SI 4 Non-zero precision for new and old PurpleAir sensors tested during 1-hour and 24-hour measurements. Results correspond to section 3.5.

	Mean		Median		Min		Max	
	Old	New	Old	New	Old	New	Old	New
Hourly rsd %	7.397	3.6418	6.99	2.7533	3.151	0.1278	42.659	46.4117
24-hour rsd %	7.201	2.6601	6.868	2.2106	5.348	0.5012	12.497	5.3627
